# Integrating Emerging Data Into Clinical Practice: A Case-Based Approach for Multiple Myeloma

**Published:** 2017-05-01

**Authors:** Sandra Kurtin

**Affiliations:** University of Arizona and Arizona Cancer Center

## Abstract

***Selected Patient Cases From the APSHO Regional Lecture Series***

**INTRODUCTION**

As the official publication of the Advanced Practitioner Society for Hematology and Oncology (APSHO), JADPRO is pleased to offer Part 2 of an accredited educational activity based on the recently concluded APSHO Regional Lecture Series. Hosted in collaboration with major cancer centers around the country, the APSHO Regional Lecture Series brought case-based didactic presentations and skills workshops to advanced practitioners.

In the spirit of JADPRO, three accredited Grand Rounds articles by Beth Eaby-Sandy, MSN, CRNP, OCN® (non–small cell lung cancer) and Sandra Kurtin, PhDc, ANP-C, AOCN® (multiple myeloma and chronic lymphocytic leukemia)—program chairs for the regional lecture series—offer the same practice-changing information and strategies for advanced practitioners.

In this Grand Rounds article, program chair Sandra Kurtin gives a comprehensive overview of the most recent developments in multiple myeloma research from the 2016 American Society of Hematology (ASH) Annual Meeting and clinical insight in the management of patients with this disease.

You can read Part 1 in the March 2017 issue of JADPRO or online at advancedpractitioner.com, and be sure to keep an eye out for Part 3 in a future issue of JADPRO. Check out apsho.org/lectures for information on registering for upcoming JADPRO Regional Lectures this year at a location near you.

**Integrating Emerging Data Into Clinical Practice: A Case-Based Approach for Multiple Myeloma**

This activity is supported by educational grants from Celgene Corporation and Janssen Biotech, Inc., administered by Janssen Scientific Affairs, LLC.

A continuing education article for nurse practitioners, clinical nurse specialists, advanced degree nurses, and oncology and hematology nurses.

**Release date:** May 9, 2017

**Expiration date:** November 13, 2017

**Expected time to complete activity:** 0.5 hour

**Meniscus Educational Institute**

3131 Princeton Pike

Building 1, Suite 205A

Lawrenceville, NJ 08648

Voice: 609-246-5000

Fax: 609-449-7969

E-mail: lrubin@harborsidemeded.com

**Journal of the Advanced Practitioner in Oncology**

94 N. Woodhull Road

Huntington, NY 11743

Voice: 631-692-0800

Fax: 631-692-0805

E-mail: claudine@harborsidepress.com

© *2017, Meniscus Educational Institute. All rights reserved.*

## Faculty

**Sandra Kurtin, PhDc, ANP-C, AOCN®,** University of Arizona and Arizona Cancer Center, Tucson, Arizona

## Activity Rationale and Purpose

The progress in the treatment of multiple myeloma has come about as a result of an increase in knowledge about its pathogenesis, the availability of novel agents developed through clinical trials, autologous stem cell transplantation, improved supportive care strategies and a better understanding of risk stratification. With the list of available therapies for multiple myeloma rapidly expanding, there is a need for increased education on the mechanism of action, toxicity profile and optimal sequencing of agents. With novel agents making their way quickly into treatment recommendations and guidelines, providers need to stay current on risk stratification strategies for multiple myeloma agents to provide patients with a balance of both efficacy and safety.

## Intended Audience

The activity’s target audience will consist of nurse practitioners, clinical nurse specialists, advanced degree nurses, and oncology and hematology nurses.

## Learning Objectives

After completing this educational activity, participants should be able to:

- Apply the principles of risk-adapted treatment using case-based scenarios to illustrate the impact of patient attributes and disease specific attributes

## Continuing Education

**Statement of Credit—Participants who successfully complete this activity (including the submission of the post-test and evaluation form) will receive a statement of credit.**

**Nurses.** This activity for 0.5 contact hour is provided by the Meniscus Educational Institute.

The Meniscus Educational Institute is accredited as a provider of continuing nursing education by the American Nurses Credentialing Center’s Commission on Accreditation.

Provider approved by the California Board of Registered Nursing, Provider No. 13164, for 0.5 contact hour.

## Financial Disclosures

All individuals in positions to control the content of this program (eg, planners, faculty, content reviewers) are expected to disclose all financial relationships with commercial interests that may have a direct bearing on the subject matter of this continuing education activity. Meniscus Educational Institute has identified and resolved all conflicts of interest in accordance with the MEI policies and procedures. Participants have the responsibility to assess the impact (if any) of the disclosed information on the educational value of the activity.

**Faculty**

**Sandra Kurtin, PhDc, ANP-C, AOCN®**, has served as a consultant for Amgen, Bristol-Myers Squibb, Celgene, Genentech, Incyte, Janssen, Novartis, Takeda, and Pharmacyclics.

**Lead Nurse Planner**

**Dorothy Caputo, MA, BSN, RN**, has nothing to disclose.

**Nurse Planner**

**Wendy J. Smith, ACNP, AOCN®**, has nothing to disclose.

**Planners**

**Jeannine Coronna** has nothing to disclose.

**Claudine Kiffer** has nothing to disclose.

**Pamela Hallquist Viale, RN, MS, CNS, ANP**, has nothing to disclose.

**Patti McLafferty** has nothing to disclose.

**Lynn Rubin** has nothing to disclose.

**Annie Yueh** has nothing to disclose.

**Content Reviewers**

**Moshe Ornstein, MD, MA**, has nothing to disclose.

## Disclaimer

This activity has been designed to provide continuing education that is focused on specific objectives. In selecting educational activities, clinicians should pay special attention to the relevance of those objectives and the application to their particular needs. The intent of all Meniscus Educational Institute educational opportunities is to provide learning that will improve patient care. Clinicians are encouraged to reflect on this activity and its applicability to their own patient population.

The opinions expressed in this activity are those of the faculty and reviewers and do not represent an endorsement by Meniscus Educational Institute of any specific therapeutics or approaches to diagnosis or patient management.

## Product Disclosure

This educational activity may contain discussion of published as well as investigational uses of agents that are not approved by the US Food and Drug Administration. For additional information about approved uses, including approved indications, contraindications, and warnings, please refer to the prescribing information for each product.

## How to Earn Credit

To access the learning assessment and evaluation form online, visit http://meded.hbrsd.com/.

**Statement of Credit:** Participants who successfully complete this activity (including scoring of a minimum of 70% on the learning assessment and complete and submit the evaluation form with an E-mail address) will be able to download a statement of credit.

## Integrating Emerging Data Into Clinical Practice: A Case-Based Approach for Multiple Myeloma

The clinical and scientific advances relative to multiple myeloma (MM) in the past decade have been staggering. Myeloma is now the 14th most common malignancy in the United States, with an estimated 30,330 new cases and 12,650 deaths in 2016 (Siegel, Miller, & Jemal, 2016). Multiple myeloma remains an incurable but highly treatable disease. There were an estimated 95,688 MM survivors in the United States in 2013, with 48.5% of them living more than 5 years (Siegel et al., 2016). In 1975, the 5-year relative survival rate for MM was 26.3% (Siegel et al., 2016). The median age at diagnosis of MM is estimated to be between 63–69 years of age. For those diagnosed between 2006 and 2013, older patients (age > 65) had inferior survival to younger patients (*p* < .01; Fonseca et al., 2016). The percentage of deaths in patients receiving treatment within 1 year of newly diagnosed MM (NDMM) decreased from 67.2% in 2006 to 21.4% in 2012, and 86.1% survived 2 years post-diagnosis in 2012 compared with 69.8% in 2006 (Fonseca et al., 2016).

The improvement in survival is attributed to treatment with novel agents and was greatest for patients treated after 2010 (Fonseca et al., 2016). Ten new agents were approved for treatment of MM between 2002 and 2016; four of them were approved in 2015 alone, and there are many new agents in clinical trials (Kazandjian & Landgren, 2016; Table 1). Among these newly approved agents are the first monoclonal antibodies and histone deacetylase inhibitors used in the treatment of MM.

**Table 1:**
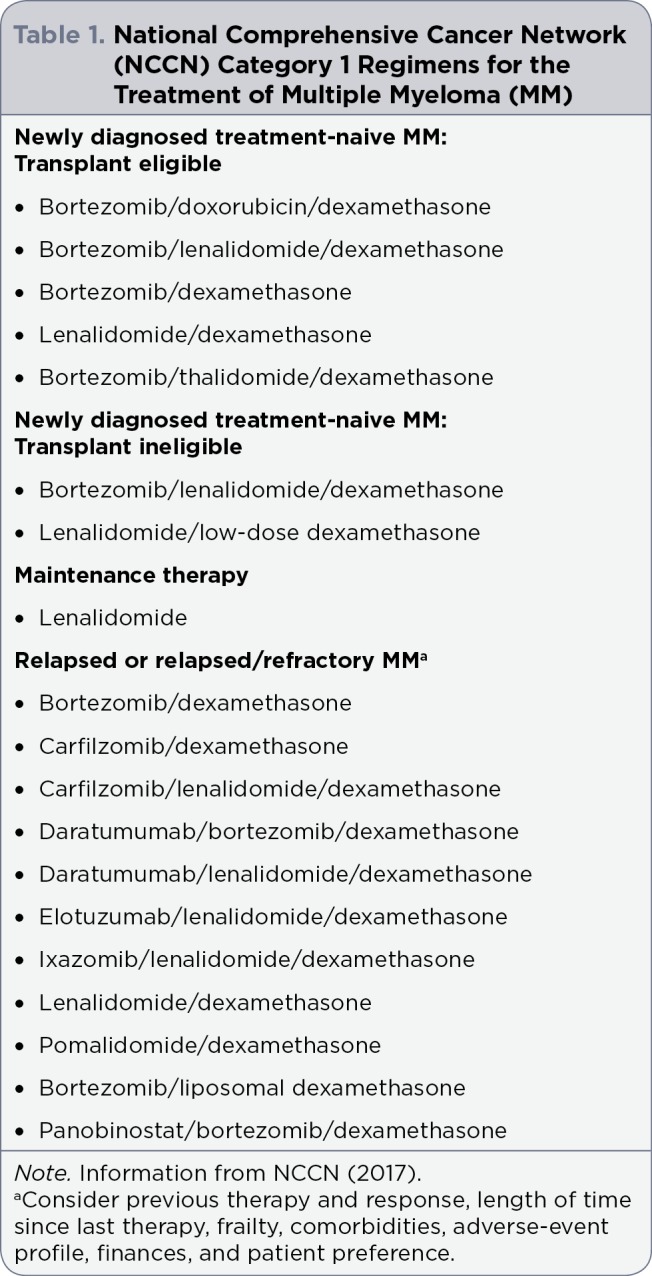
National Comprehensive Cancer Network (NCCN) Category 1 Regimens for the Treatment of Multiple Myeloma (MM)

Improved understanding of the pathobiology of MM has improved identification of novel targets and pathways, which can be exploited for therapeutic benefit. In addition, strategies for risk-adapted treatment selection, enhanced diagnostic technologies, techniques for mitigating disease and treatment-related adverse events, and improvements in palliative and supportive care have enhanced patient outcomes. None of this would be possible without continued clinical trials.

The robust pace of discovery is reflected in the many updated or newly developed practice guidelines (Table 2). A PubMed search using the terms [multiple myeloma] AND [treatment] with adults, English, humans, and the year 2016 retrieved 163 peer-reviewed journal articles. More than 200 abstracts relevant to the diagnosis and treatment of multiple myeloma were presented at the American Society of Hematology (ASH) Annual Meeting in December 2016. Collectively, this almost frenetic pace of discovery and dissemination of clinical and scientific data presents a challenge to hematology/oncology clinicians, MM patients, and advocacy organizations. Considering that MM represents only 1.8% of new cancer diagnoses (Siegel et al., 2016), the challenge to assimilate and apply this information is particularly arduous for health-care providers who do not specialize in MM or even hematologic malignancies. The goal of this article is to summarize some of the key findings from the ASH Annual Meeting and other recently published literature using MM cases to illustrate application.

**Table 2:**
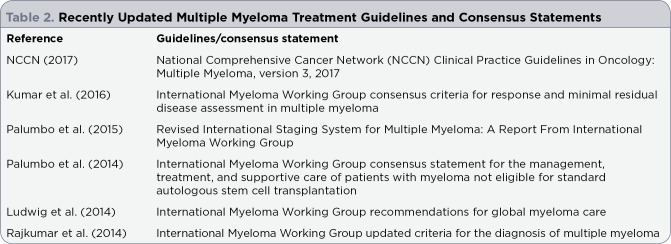
Recently Updated Multiple Myeloma Treatment Guidelines and Consensus Statements

## SMOLDERING AND ASYMPTOMATIC MULTIPLE MYELOMA

**Case Study 1**

Mr. G, a 69-year-old male, presented to his primary care provider with complaints of progressive fatigue and low-back pain over a 6-month period. He had never had any major illnesses and takes no prescription medicines. He is an avid bicyclist but has not been able to ride due to his symptoms.

Mr. G’s physical exam was unremarkable, except for a slow gait due to his back pain. Initial laboratory results showed no significant abnormalities, except for an elevated total protein level (9.1 g/dL; range 6.4–8.3 g/dL), mild anemia (11.9 g/dL; range 13.5–17.5 g/dL), and borderline renal insufficiency (creatinine clearance of 50 mL/min, normal ≥ 60 mL/min). Plain films of the lumbar and sacral spine were obtained, showing degenerative changes and a compression deformity at the L2 vertebra. A magnetic resonance imaging (MRI) of the spine was ordered, showing soft-tissue nodules within the T12, L2, and L3 vertebrae. Additional laboratory testing was obtained, showing an elevated immunoglobulin G (IgG) level (3.2 g/dL). Serum protein electrophoresis (SPEP) and serum free light chain (SFLC) assays showed a kappa-restricted monoclonal IgG protein and an elevated kappa light chain level (934.4 mg/L; range 3.3–19.4 mg/L) and a kappa/lambda ratio (κ:λ) of 60.83 (range 0.26–1.65). A bone marrow biopsy was performed, showing 12% kappa-restricted plasma cells.

**Discussion**

Prior to the updated guidelines proposed by the International Myeloma Working Group (IMWG), Mr. G would be classified as having smoldering multiple myeloma (SMM; Rajkumar et al., 2014). The presence of at least one lesion on MRI, along with the diagnostic criteria for SMM, changes the diagnosis to MM. Myeloma-defining events (MDEs) incorporate the previous CRAB criteria (hypercalcemia, renal dysfunction, anemia, and bone lesions), denoting the presence of end-organ damage but adding biomarkers that represent an 80% risk of progression to active myeloma within 2 years (Rajkumar, Landgren, & Mateos, 2015). These biomarkers include the presence of > 1 lesion on MRI, clonal bone marrow plasma cells (BMPC) > 60%, or an involved to uninvolved SFLC ratio ≥ 100 (Rajkumar et al., 2014). Close monitoring for attributes now considered to be MDEs will allow for early diagnosis of active MM and prevention of end-organ damage (Rajkumar et al., 2015).

The approach to treatment for patients with high-risk SMM is less clear. Patients with high-risk features should be monitored closely. An increase in the serum monoclonal protein level by at least 10% on two successive evaluations within a 6-month period has been associated with a 65% probability of disease progression in SMM (Sundararajan, Kumar, Korde, & Agarwal, 2016). Speciﬁc cytogenetic abnormalities, especially translocation t(4;14), 1q gain, and deletion 17p, have been associated with a high risk of disease progression (Sundararajan et al., 2016). Non-IgG, in particular IgA SMM and the presence of immunoparesis, is an additional feature that portends higher-risk disease (Leng & Lentzsch, 2016).

The Mayo Clinic risk model for SMM cites three factors associated with higher-risk disease: 1) M-protein ≥ 3g/dL; 2) BMPC ≥ 10%; and 3) a κ:λ FLC of either ≤ 0.1255 or ≥ 8. These attributes are associated with a median time to progression (TTP) to active MM of 1.9 years (Kyle et al., 2007).

Two abstracts presented at the 2016 ASH Annual Meeting emphasize the role of clinical trials in areas where there are not yet approved therapies. Mateos et al. (2016) randomized 119 high-risk SMM patients to 4 cycles of lenalidomide (Revlimid; 25 mg, 21/28 days) and dexamethasone (20 mg/day on days 1–4 and days 12–15), followed by maintenance (lenalidomide at a dose of 10 mg/day on days 1–21 of each 28-day cycle) for up to 2 years vs. observation (current standard of care). The patients receiving treatment with lenalidomide/dexamethasone experienced a 57% reduction in the risk of death (hazard ratio [HR], 0.43; 95% confidence interval [CI] = 0.2–0.9; *p* = .02) at a median follow-up of 75 months (range: 57–100 months), compared with the observation arm.

Additionally, there is a trend toward improved overall survival (OS) in the treatment arm (86%) vs. the observation arm (62%) at 72 months, although the median OS has not been reached in either group. More patients in the observation arm progressed to active MM (86%, n = 53/62) vs. the treatment arm (38%, n = 22/57). At the time of disease progression, most patients were treated with novel agents, with response rates similar to those seen in trials for NDMM. The survival benefit observed was independent of the classification model used for defining high-risk SMM (Mateos et al., 2016).

A second abstract with the objective of determining progression-free survival (PFS) from high-risk SMM to symptomatic MM evaluated the standard dosing combination of lenalidomide/dexamethasone plus elotuzumab (Empliciti; Ghobrial et al., 2016). A subset of older patients (age > 65) with high-risk cytogenetics were randomized to receive low-dose dexamethasone (20 mg weekly). Both groups were given the option to collect stem cells after 8 cycles or best response and were then treated with maintenance therapy consisting of elotuzumab (20 mg/kg) on day 1, in combination with lenalidomide on days 1–21 of a 28-day cycle. A total of 34 of 39 patients were evaluable, with an overall response rate (ORR) of 71%, including 9 very good partial responses (VGPR; 26%) and 15 partial responses (PR; 44%), with no patients showing disease progression.

The treatment was well tolerated, with no grade 4 toxicity and low rates of grade 3 toxicity: hypophosphatemia (23%), neutropenia (8%), infection (8%), anemia (3%), pulmonary embolism (3%), rash (3%), and diarrhea (3%). Although these trials provide promising results, with consideration of cost, time required for treatment, and potential for end-organ damage due to treatment in a population that by definition does not yet have this disease, recommendations are to fully evaluate patients with SMM and continue to enroll them into clinical trials to more fully evaluate risk and benefit (Leng & Lentzsch, 2016).

## RISK-ADAPTED TREATMENT SELECTION

Despite all the progress made, perhaps one of the greatest challenges is the simple fact that MM is not one disease (Lonial & Nooka, 2016). Rather, MM is a group of heterogeneous plasma cell malignancies with a continuum characterized by variable tempos with intervals of response and relapse (Figure ). Until very recently, MM has been considered a highly treatable but incurable disease (Rajkumar & Kumar, 2016). Today, there is hope that in selected NDMM patients who achieve a rapid, deep, and sustained response with no evidence of minimal residual disease (MRD–), sustained over a period of years, a cure may be possible (Barlogie et al., 2014; Katodritou, Papadaki, Konstantinidou, & Terpos, 2016; Salem & Ghobrial, 2015). Achieving these outcomes requires emulation of clinical trials and tailoring of therapy for the individual patient. The IMWG has recently updated the response criteria to incorporate parameters for MRD status (Kumar et al., 2016). Let’s look at some cases to illustrate these concepts.

**Figure:**
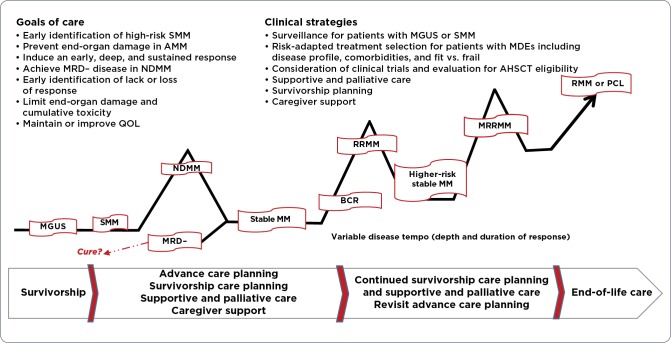
Multiple myeloma continuum of care. SMM = smoldering multiple myeloma; AMM = asymptomatic multiple myeloma; MRD– = minimal residual disease–negative; NDMM = newly diagnosed multiple myeloma; QOL = quality of life; MGUS = monoclonal gammopathy of uncertain significance; MDEs = myeloma- defining events; AHSCT = autologous hematopoietic stem cell transplantation; = transition point; BCR = biochemical relapse; RRMM = relapsed/refractory multiple myeloma; MRRMM = multiple relapsed/refractory multiple myeloma; RMM = refractory multiple myeloma; PCL = plasma cell leukemia.

**Case Study 2a**

A 47-year-old divorced female presents with IgA lambda NDMM, revised International Staging System (R-ISS) stage II, with t(11;14), a compression fracture at the T12 vertebra, and widespread lytic lesions. Comorbidities are poorly controlled insulin-dependent diabetes, obesity, sedentary lifestyle, current smoker, diabetic neuropathy, and a history of a provoked deep-vein thrombosis following hysterectomy. The patient lives alone and is currently on disability.

**Case Study 2b**

A 64-year-old male presents with IgG kappa NDMM, R-ISS stage III (lactate dehydrogenase [LDH] > upper limit of normal [ULN]), fluorescence in situ hybridization (FISH)-detected t(14;16), del(17p), and hyperdiploidy. Comorbidities are atrial fibrillation, coronary artery disease with a three-vessel bypass, congestive heart failure, and chronic renal insufficiency (creatinine clearance [CrCl] = 40 mL/min). The patient lives with his wife and has two adult children and three grandchildren living out of town.

**Case Study 2c**

A 70-year-old male presents with NDMM, kappa light chain disease. He has FISH-detected t(4;14), with normal albumin, #x00e2;2 microglobulin, and LDH. He has R-ISS stage I disease. Comorbidities are seasonal allergies and benign prostatic hypertrophy. The patient is an avid tennis player and lives with his partner in an active adult retirement community. The patient has no children.

**Discussion**

These cases illustrate the diversity of patients with NDMM. Risk stratification in treatment selection requires consideration of MM attributes as well as the attributes of the individual patient. The first step is to fully characterize each patient’s disease and establish transplant eligibility.

When considering transplant eligibility, the first consideration in most health-care professionals’ minds is age. However, comorbidities, fitness, and caregiver support are critical considerations. Patient 2a, although young, has complex and poorly controlled comorbidities and continues to engage in habits detrimental to her health. In addition, she has limited support. She would not be an ideal candidate for a stem cell transplant (SCT). On the other hand, patient 2c, although at the upper limit of age for SCT, is in good physical health other than the MM and has excellent support. Patient 2b could also be considered for SCT; however, he will require careful monitoring of his heart disease to avoid adverse events that may exclude this treatment option. Based on the elevated LDH at baseline, he is considered to have higher-risk disease (Yuan et al., 2016).

Each of these patients has attributes of disease that hold prognostic and clinical significance. Patient 2a is young and has low-risk disease; however, given her general health and social situation, she is not an ideal candidate for SCT.

Patient 2b has high-risk disease based on the t(14;16) and del(17p), both representing high-risk disease. He has a significant cardiac history and renal insufficiency, which will need consideration in selecting his treatment, but he would be eligible for SCT. He also has an LDH that is above the ULN.

Elevated serum LDH is a new addition to risk stratification for MM, recently added to the R-ISS (Palumbo et al., 2015). In a study of 107 older (age > 60 years) MM patients, LDH level correlated with median OS (52.5 ± 6.9 months in normal LDH group, 15.5 ± 5.2 months in elevated LDH group [*p* < .001]). Similarly, median PFS differed in the two groups (24.0 ± 3.5 months in normal LDH, 12.0 ± 10.5 months in elevated LDH group [p = .008]). Multiple factors analysis showed that LDH was an independent prognostic factor of elderly MM, validating the addition of this measure to the R-ISS. Median OS based on R-ISS varies by stage with the addition of LDH and FISH high-risk features (Jimenez-Zepeda et al., 2016; Palumbo et al., 2015; Yuan et al., 2016). Importantly, the ULN for LDH varies by individual lab and must be evaluated within this context.

Patient 2c, the oldest in the group, is fit, yet he carries the t(4;14), which places him in a higher-risk group and has clinical implications, namely the use of bortezomib (Velcade)-based therapies (Weinhold et al., 2016). He has good social support and would be an ideal candidate for SCT. Chronologic age, although important, should not be the only consideration for transplant eligibility. The t(4;14) implies intermediate risk with implications for not only induction therapy, but perhaps maintenance therapy (Lipe, Vukas, & Mikhael, 2016).

## RELAPSED, RELAPSED/REFRACTORY, AND REFRACTORY MULTIPLE MYELOMA

Relapse is inevitable for most patients with MM. The time to relapse is highly variable; however, each relapse is characterized by a shorter duration and lower depth of response, explaining the emphasis on maximizing outcomes in NDMM (Figure). Fortunately, achievements in the basic science and clinical trials enrollment that have generated results leading to approval of new therapies, including many with novel mechanisms of action, hold great promise for the patient with relapsed/refractory MM (RRMM; Table 1). As with most cancer trials, novel agents must be tested in the relapsed or relapsed/refractory setting. Once approved, newer agents are studied in combination and in earlier-stage disease. This paradigm is clear in the recent trials in MM, some of which have practice-changing implications.

Perhaps one of the most exciting developments is the introduction of monoclonal antibodies into the treatment of MM. Like the changes to the natural history of non-Hodgkin lymphoma, with the introduction of anti-CD20 monoclonal antibodies (MoAbs), there is hope that MoAbs represent a therapeutic class in MM, with novel targets and without overlapping adverse events and toxicity profiles compared with established MM agents. Monoclonal antibodies that are used across diagnoses are associated with hypersensitivity reaction (Palumbo et al., 2016; Thanendrarajan et al., 2016; Weisel, 2016). Premedication, close monitoring for the first infusions, and prompt interventions for signs and symptoms of hypersensitivity reactions have been proven to effectively mitigate the severity of these reactions and, in most cases, allow for continued treatment (Boudreault, Touzeau, & Moreau, 2017; Colson, 2015; Costello, 2017; Gallimore, 2016; Hofmeister & Lonial, 2016).

What is unknown now are the long-term outcomes in patients treated with these novel agents. Importantly, daratumumab (Darzalex), an anti-CD38 antibody, has activity as a single agent or in combination with other novel agents and has been studied in patients receiving multiple lines of therapy (Costello, 2017; Palumbo et al., 2016). A phase III trial randomizing 498 patients with relapsed or relapsed and refractory multiple myeloma (CASTOR trial) to receive standard-dose bortezomib/dexamethasone alone vs. bortezomib/dexamethasone in combination with daratumumab showed significantly improved PFS (7.16 months without daratumumab vs. not reached with daratumumab, *p* < .0001), TTP (7.29 months vs. not reached, respectively, *p* < .0001), and ORR (63% vs. 83%, respectively, p < .0001). Infusion reactions did occur with daratumumab; most were reported during the first infusion and were grade 1 to 2.

A phase III study (POLLUX trial) evaluating daratumumab in combination with lenalidomide and dexamethasone randomized 569 MM patients to receive daratumumab, lenalidomide, and dexamethasone or lenalidomide and dexamethasone alone. The triple-drug combination was associated with a 63% reduction in the risk of disease progression or death at 13.5 months of follow-up (p*p* < .0001; Dimopoulos et al., 2016). Addition of daratumumab also significantly increased ORR compared with lenalidomide and dexamethasone alone: complete response (CR) rates or better were 43% vs. 19%, respectively (*p* < .0001), whereas VGPR rates or better were seen in 76% vs. 44%, respectively (*p* < .0001; Dimopoulos et al., 2016). No significant differences in adverse-event profiles compared with established trials were noted.

The primary difficulty in administering daratumumab-containing regimens is the time required for infusion. Subcutaneous (SC) delivery of daratumumab is being tested in combination with the recombinant human hyaluronidase enzyme (rHuPH20) to facilitate systemic absorption of the SC infusion into the abdominal wall.

The PAVO study evaluated two doses of daratumumab (DARA)-PH20 to determine which dose would achieve systemic concentrations most similar to that which is achieved with intravenous (IV) administration (Usmani et al., 2016). The 1,800-mg dose given in 90 mL over 30 min, via a syringe pump at rotating sites on the abdomen, was selected for phase II of this study based on tolerability and efficacy in this highly pretreated population (n = 17, median of 4 prior lines of therapy [range 2–7]; prior autologous stem cell transplant [ASCT], 76%; proteasome inhibitor [PI]–refractory only, 6%; immunomodulatory drug [IMiD]–refractory only, 12%; double refractory to PI and IMiD, 65%). The toxicity profile was like that of IV daratumumab. The ORR rate was 41% (3 VGPRs, 4 PRs), with a median time to response of 4 weeks (range 4–8 weeks).

Elotuzumab, which has a very different mechanism of action, targeting the signaling lymphocytic activation molecule family member 7 (SLAMF7) and inducing natural killer cell death and antibody-dependent cell-mediated cytotoxicity, is not active as a single agent but holds great promise in combination with other novel agents by activating elements of the immune response and microenvironment (Boudreault et al., 2017; Weisel, 2016). Combination trials using elotuzumab with lenalidomide and dexamethasone in SMM have been recently published (Ghobrial et al., 2016).

A phase II trial randomized 150 RRMM patients to elotuzumab, bortezomib, and dexamethasone (EBd) vs. bortezomib and dexamethasone (Bd, n = 77) or Bd (n = 75). Very good partial response or better occurred in 36% of patients (EBd) vs. 27% (Bd). In patients with RRMM, elotuzumab appears to provide clinical benefit without added clinically significant toxicity when combined with Bd vs. Bd alone. Discontinuation of therapy in the overall population was mostly due to disease progression (57%; Jakubowiak et al., 2016). It is conceivable that we will see combinations of novel agents with MoAbs moving into the front-line setting (Hofmeister & Lonial, 2016). In addition, there are several monoclonal antibodies, immune-mediating agents, and targeted therapies currently in clinical trials (Table 3).

**Table 3:**
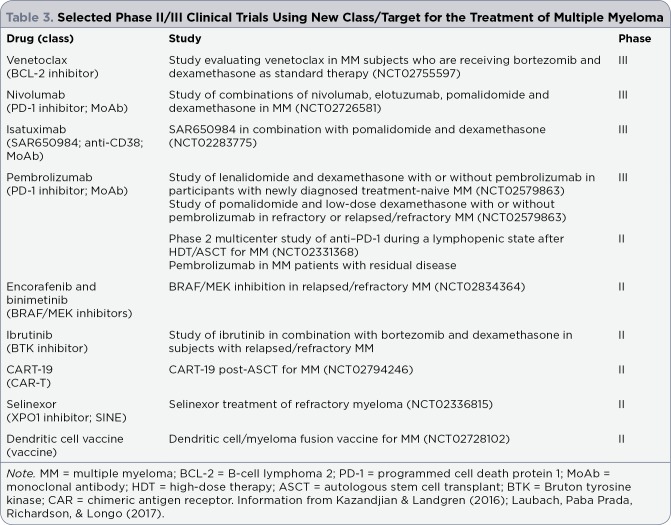
Selected Phase II/III Clinical Trials Using New Class/Target for the Treatment of Multiple Myeloma

## THE ROAD AHEAD

It is widely accepted that MM is a multiclonal malignancy at the time of diagnosis with intraclonal variability across individual patients (Brioli, Melchor, Cavo, & Morgan, 2014; Keats et al., 2012). Therefore, the MM profile for an individual patient will likely vary over time with acquired genetic instability (Manier et al., 2016; Shay, Hazlehurst, & Lynch, 2016). Investigating the effect of therapeutic intervention on genetic instability, particularly with alkylating agents, will be needed.

For many patients, multiple pathways are deregulated, mutation prevalence increases with disease progression, and the number of adverse markers has an additive effect on OS (Boyd et al., 2012; Kuiper et al., 2015). Gene-expression profiling with mutational testing, evaluation of MRD– status with a goal to achieve 10^-6^ depth of response using 8 color flow cytometry on bone marrow samples, and continued tandem tissue banking in conjunction with therapeutic trials will be necessary to continue to find actionable targets, identify patients who will benefit most from treatment, and make the science count for these patients. Offering advance care planning, supportive and palliative care, and caregiver support remain critical to quality of life and balancing the promise of science with the complex and long-term treatment necessary for the patient with MM.
